# Iquitos Virus: A Novel Reassortant *Orthobunyavirus* Associated with Human Illness in Peru

**DOI:** 10.1371/journal.pntd.0001315

**Published:** 2011-09-20

**Authors:** Patricia V. Aguilar, Alan D. Barrett, Mohammad F. Saeed, Douglas M. Watts, Kevin Russell, Carolina Guevara, Julia S. Ampuero, Luis Suarez, Manuel Cespedes, Joel M. Montgomery, Eric S. Halsey, Tadeusz J. Kochel

**Affiliations:** 1 U.S. Naval Medical Research Unit Six, Lima, Peru; 2 Department of Pathology, University of Texas Medical Branch, Galveston, Texas, United States of America; 3 Directorate of Epidemiology, Lima, Peru; 4 National Institute of Health, Lima, Peru; USAMRIID, United States of America

## Abstract

Oropouche (ORO) virus, a member of the Simbu serogroup, is one of the few human pathogens in the *Orthobunyavirus* genus in the family *Bunyaviridae*. Genetic analyses of ORO-like strains from Iquitos, Peru, identified a novel reassortant containing the S and L segments of ORO virus and the M segment of a novel Simbu serogroup virus. This new pathogen, which we named Iquitos (IQT) virus, was first isolated during 1999 from a febrile patient in Iquitos, an Amazonian city in Peru. Subsequently, the virus was identified as the cause of outbreaks of “Oropouche fever” during 2005 and 2006 in Iquitos. In addition to the identification of 17 isolates of IQT virus between 1999 and 2006, surveys for neutralizing antibody among Iquitos residents revealed prevalence rates of 14.9% for ORO virus and 15.4% for IQT virus. Limited studies indicate that prior infection with ORO virus does not seem to protect against disease caused with the IQT virus infection. Identification of a new *Orthobunyavirus* human pathogen in the Amazon region of Peru highlights the need for strengthening surveillance activities and laboratory capabilities, and investigating the emergence of new pathogens in tropical regions of South America.

## Author Summary

Oropouche (ORO) virus is one of the few human pathogens in the *Orthobunyavirus* genus in the family *Bunyaviridae*. Phylogenetic analyses of ORO-like strains isolated from febrile patients in Iquitos, Peru, identified a novel ORO reassortant virus, which we named Iquitos (IQT) virus based on the location of the isolation of the virus. This novel pathogen was first isolated during 1999 from a 13-year-old boy who had an illness that included symptoms of fever, headache, eye pain, body pain, arthralgias, diarrhea, and chills. Subsequently, the virus was identified as the cause of outbreaks of “Oropouche fever” during 2005 and 2006 in Iquitos. Limited serological studies indicate that prior infection with ORO virus does not seem to protect against disease caused with the IQT virus infection. In summary, we identified a new *Orthobunyavirus* human pathogen in the Amazon region of Peru; these results highlight the need for strengthening surveillance activities and investigating the emergence of new pathogens in tropical regions of South America.

## Introduction

Viruses in the family *Bunyaviridae* are classified into five genera: *Orthobunyavirus, Hantavirus, Nairovirus, Phlebovirus* and *Tospoviruses*. The orthobunyaviruses are enveloped, negative sense RNA viruses whose genome comprises three segments: small (S), medium (M) and large (L). The S segment encodes for the nucleocapsid and a nonstructural protein, NSs. The M segment encodes for the glycoproteins Gn and Gc, whereas the L segment encodes the viral polymerase. Some members of the *Orthobunyavirus* genus are known to cause clinical disease in humans, including Oropouche (ORO) virus, a member of the Simbu serogroup, which causes a febrile disease often associated with headache, dizziness, weakness, myalgias and arthralgias [1]. Bunyamwera virus, which is considered the prototype member of the family, causes a febrile illness with headache, arthralgias, rash and infrequent central nervous system involvement [2]. Hemorrhagic manifestations associated with some orthobunyavirus infections have also been reported recently [3], [4].

Genetic reassortment among members of the same serogroup within the *Orthobunyavirus* genus occurs in nature and has led to the emergence of new viruses, occasionally with increased pathogenicity. This appears to be the case with Ngari virus, which has been associated with hemorrhagic fever in Kenya and Somalia [3], [5]. This virus is comprised of the S and L segments of Bunyamwera virus and the M segment of Batai virus [4]. On the basis of genetic and antigenic analyses, we previously reported that Jatobal virus (JAT), a member of the Simbu serogroup, is a reassortant virus that contains the S segment of ORO virus and the M and L segments of a still unrecognized Simbu serogroup virus [6].

Within the *Orthobunyavirus* genus, 18 serogroups have been recognized on the basis of the results of cross-hemagglutination inhibition (HAI) and antibody neutralization relationships [7]. The Simbu serogroup contains at least 25 members, and recent phylogenetic analyses demonstrated that the genetic relationships amongst these viruses are consistent with the results of serological relationships [7], [8]. ORO virus was originally isolated in 1955 from blood of a febrile forest worker who lived in Vega de Oropouche, Trinidad [9]. Outbreaks involving thousands of human cases continue to be reported in Brazil [10], [11], [12], [13], and circulation of the virus has been reported in Panama, Peru, and Trinidad [14], [15], [16], [17]. Based on the S segment, three genotypes of ORO virus were distinguished phylogenetically: genotype I includes the isolates from Brazil and Trinidad, genotype II includes isolates from Brazil and Peru, and genotype III is represented by the isolates from Brazil and Panama [18], [19].

ORO virus has been isolated from mosquitoes (*Coquillettidia venezuelensis* in Trinidad [9]; *Ochlerotatus serratus* and *Culex quinquefasciatus* in Brazil [20]) and frequently from the midge *Culicoides paraensis* [9], [21], [22]. High population densities of this midge have been found during epidemics of ORO virus [23]. Transmission of the virus has been demonstrated via *C. paraensis*, from infected to susceptible hamsters, and from infected humans to susceptible hamsters [20], [24]. ORO virus has also been isolated from sloths (*Bradypus tridactylus* [20]), and from a monkey (*Callithrix sp*.) in Brazil [10].

In 1995, the U.S. Naval Medical Research Unit Six (NAMRU-6) in Lima, in collaboration with the Ministry of Health of Peru, initiated a passive surveillance study to investigate etiology of febrile illnesses. As part of these surveillance activities, several ORO-like strains were obtained from febrile patients in Iquitos, Peru. In this study, we describe the identification of a novel reassortant virus which we named Iquitos (IQT) based on the location of the isolation of the virus. Specifically, we: 1) demonstrated that IQT virus causes clinical disease in humans similar to ORO fever, 2) described the genetic relationship of IQT virus to other members of the Simbu serogroup, 3) identified the risk factors associated with human infection by IQT virus in an urban setting of the Amazon region of Peru, and 4) describe the clinical manifestations associated with infection. Significantly, immunity to ORO virus does not appear to protect against infection by IQT virus, and both ORO and IQT viruses appear to have similar antibody prevalence in Iquitos over the past 10 years.

## Materials and Methods

### Viruses

The IQT1690 ORO strain used in this study was isolated in 1995 from a 50 year-old male resident of Iquitos, Peru. The first strain of IQT virus (IQT9924) was isolated in Iquitos from a 13 year-old boy (Table 1). The origin of the ORO strains BeH379693, BeH544552, MD023, GML444477 and GML 445252 were previously described [18], [19].

**Table 1:** Viremia titer for human febrile cases in Iquitos, Peru, included in the study.

CODE	COLLECTION DATE	ONSET	AGE	GENDER	Viremia (PFU/ml)	Virus
**IQT1690**	6 Apr-95	1 Apr-95	50	Male	7×10^5^	ORO
**IQT7085**	6 Apr-98	5 Apr-98	28	Male	6×10^3^	ORO
**IQT9924**	12 Feb-99	10 Feb-99	13	Male	<100	IQT
**IQD5900**	25 Jun-03	22 Jun-03	34	Female	1.2×10^4^	IQT
**IQE1005**	30 Mar-05	29 Mar-05	30	Male	<100	IQT
**IQE1217**	25 Apr-05	19 Apr-05	24	Male	1.8×10^5^	IQT
**IQE1283**	3 May-05	1 May-05	30	Female	4.4×10^3^	IQT
**IQE1704**	6 Jul-05	5 Jul-05	27	Female	<100	IQT
**IQE1816**	19 Jul-05	17 Jul-05	15	Female	1.2×10^4^	IQT
**IQE1819**	21 Jul-05	17 Jul-05	40	Male	1.8×10^4^	IQT
**IQE2257**	2 Nov-05	1 Nov-05	22	Male	7.6×10^4^	IQT
**IQE2718**	19 Jan-06	18 Jan-06	21	Male	4.2×10^4^	IQT
**IQE2791**	27 Jan-06	24 Jan-06	32	Female	6×10^2^	IQT
**IQE2803**	30 Jan-06	29 Jan-06	28	Male	2×10^2^	IQT
**IQE2973**	13 Feb-06	12 Feb-06	15	Male	1.8×10^4^	IQT
**IQE3173**	27 Feb-06	26 Feb-06	55	Female	2.2×10^4^	IQT
**IQE3248**	3 Mar-06	1 Mar-06	21	Female	6×10^4^	IQT
**IQE3728**	21 Apr-06	20 Apr-06	25	Male	<100	IQT
**NFI0309**	23 Feb-06	21 Feb-06	35	Male	3×10^4^	IQT

### Study site

Iquitos is a city of about 380,000 inhabitants located 120 meters above sea level in the Amazon Basin in northeastern Peru.

### Febrile surveillance study population

The human use study protocols were approved by the Ministries of Health of Peru and by the NAMRU-6 Institutional Review Board (protocol NMRCD.2000.0006). The study subjects were patients (≥ 5 years of age) who presented with a diagnosis of an acute, febrile undifferentiated illness at military or civilian outpatient clinics in Iquitos. The criteria for inclusion in the program was fever ≥ 38°C of no more than five days duration, headache, myalgia and other nonspecific symptoms. Demographic and clinical information were obtained from each patient at the time of voluntary enrollment and a signed consent form was obtained from patients 18 years of age and older. In addition, written assent was obtained from patients between 8 and 17 years of age. For patients younger than 18 years, written consent was obtained from parents or legal guardians. Paired-blood samples were collected, one during the acute phase of illness and a second sample 2-4 weeks after onset of symptoms.

### Virus isolation and serological assay

Acute serum samples were tested for virus by cell culture, and both acute and convalescent serum samples were assayed for IgM antibody to a variety of arboviruses by an enzyme-linked immunosorbent assay (ELISA), as described previously [25].

For virus isolation attempts, serum samples were diluted 1∶5 in Eagle's minimum essential medium (EMEM) supplemented with 2% fetal bovine serum, 200 µg of streptomycin, and 200U/ml of penicillin. Two hundred µl of the diluted samples were inoculated into flasks containing either confluent monolayers of African green monkey kidney (Vero) cells or *Aedes albopictus* mosquito (C6/36) cells. Vero and C6/36 cell cultures were maintained at 37°C and 28°C, respectively, and were examined daily for 10 days for evidence of viral cytopathic effects (CPE). Spot-slides of C6/36 and Vero cells were subsequently prepared on days 10 post-inoculation of the samples (or sooner if CPE developed) and an immunofluorescence assay (IFA) was performed using polyclonal antibody against arboviruses endemic to Peru, including ORO virus [17], [25], [26], [27], [28], [29], [30].

### Neutralizing antibody prevalence studies

A total of 1037 human serum samples collected in Iquitos during 2006 were tested for IgG antibody to the ORO strain IQT1690 and IQT9924 virus using a previously described ELISA [25]. The samples were collected as part of a cross-sectional antibody prevalence study conducted in Iquitos after an outbreak of febrile illness associated with Venezuelan equine encephalitis virus (VEEV) infection (protocol PJT.NMRCD.001). Three neighborhoods where VEE cases were reported and a control neighborhood where VEE cases were not reported were included in the study [31]. Serum samples from a subset of the original study participants who agreed to future use of their samples were tested by ELISA IgG antibody. ELISA positive samples were further tested using an 80% plaque reduction neutralization assay (PRNT) each for the ORO strain IQT 1690 and IQT9924 virus. Briefly, sera were heat-inactivated at 56°C for 30 minutes and 2-fold serum dilutions were prepared and mixed with 100 plaque forming units (PFUs) of each virus and incubated at 4°C overnight. The virus-serum dilutions mixtures were inoculated into confluent monolayer of Vero cells propagated in microplates and incubated at 37°C for 1 hour before adding an overlay of 0.4% of agarose in EMEM. After 72 hours of incubation at 37°C, the plates were stained with 0.25% crystal violet in 20% methanol and plaques were counted. All IgG antibody positive samples were tested at an initial concentration of 1∶20 and all positive samples were further titrated to determine endpoint titers. Neutralization titers were considered as the highest serum dilution that reduced plaque formation by ≥ 80%.

### Extraction of RNA, RT and PCR amplification of S, M and L segments

Viral RNA was extracted using the QIAamp viral RNA mini kit (Qiagen, Valencia, CA) or Trizol reagent (Invitrogen, Carlsbad, CA) following the manufacturer's protocols. The reverse transcription reaction (RT) was done using 1X RT buffer, 0.2 mM dNTPs, 1 µM of primers, 80 units of RNAsin ribonuclease inhibitor (Promega, Madison, WI), 1mM of dithiothreitol, 200U of SuperScript reverse transcriptase (Invitrogen) and 5 µl of RNA. The reactions were incubated at 42°C for 1 hour. The PCR included 1X PCR buffer, 0.25 mM dNTPs, 1 µM of primers, 3 mM of MgCl_2_, 2.5 units of GoTaq DNA polymerase (Promega, Madison, WI) and 5 µl of cDNA. The conditions for the PCRs included incubation at 94°C for 2 minutes, 35 cycles of 94°C for 30 seconds, 50°C for 1 minute, 72°C for 1.5 minutes and a final extension of 72°C for 10 minutes to ensure complete double-stranded DNA synthesis. The primers used for the PCR amplification have been previously described and included ORO N3 (GTGAATTCCCACTATATGCCAATTCCGAATT) and ORO N5 (AAAGAGGATCCAATAATGTCAGAGTTCATTT) that amplifies the S segment, M14C (CGG AAT TCA GTA GTG TAC TACC) and M619R (GAC ATA TG (CT) TGA TTG AAG CAA GCA TG) that amplifies the M segment and M13CBunL1C (TGTAAAACGACGGCCAGTAGTGTACTCCT), and BunL605R (AGTGAAGTCICCATGTGC) that amplifies the L segment [3], [10], [19].

### Sequencing and phylogenetic analyses

Partial sequences of the S, M, and L segments were obtained and compared to published ORO virus sequences. Purified PCR products were sequenced directly and sequencing analyses of the PCR products were performed using an Applied Biosystems Prism automated DNA sequencing kit (Foster City, CA) according to the manufacturer’s’ protocols. Sequences were aligned using the Clustal program in the Mac Vector software package (MacVector Inc., Cary, NC) and phylogenetic analyses were performed using the maximum parsimony, neighbor joining, and maximum likelihood methods implemented in the PAUP software [32], [33]. For the neighbor joining analyses, the HKY85 distance was used. Bootstrap values, to place confidence values on grouping within trees, were calculated based on 500–1000 replicates.

### Serological reagents and antigenic characterization of ORO-like viruses

To prepare ORO virus hyperimmune ascitic fluid for classical cross-neutralization tests, mice received 4 weekly intraperitoneal (IP) injections of virus-infected newborn mouse brain suspension with Freud's adjuvant. To investigate antigenic differences among the viruses, cross-neutralization assays were then performed, using a previously described PRNT [34]. To further evaluate the antigenic differences between the ORO strain IQT1690 and IQT9924 virus, acute and convalescent sera from patients infected with these viruses were tested against both strains using PRNT.

### Hamster lethal dose 50 (LD_50_) studies

Three to 4 week-old golden Syrian female hamsters were inoculated (IP) with serial 10-fold dilutions of virus. The LD_50_ was calculated by the Reed and Muench method [35].

### Viremia levels

To determine viremia levels in infected patients, serum samples were prepared in 10-fold dilutions and 100 µl of each dilution was inoculated onto confluent monolayers of Vero cells in 12 well-plates. Viruses were adsorbed to the monolayers for 1 hour at 37°C. A 3-ml overlay consisting of EMEM with 0.4% agarose was added, and the cells were incubated at 37°C for 72 hours. Agar plugs were removed, and the cells were stained with 0.25% crystal violet in 20% methanol. The sensitivity of the assay corresponded to a detection limit of 100 PFU/ml.

### Statistical analyses

The analysis was performed using SPSS 17.0 for Windows (SPSS Inc, Chicago, IL, USA). The proportions of positive results (PRNT for IQT1690 and IQT9924 antibody ≥ 20) were calculated with their respective 95% confidence intervals (95% CI). The proportions were compared using the Pearson Chi-Square and Fisher exact tests. A 2-tailed p-values < 0.05 was used for all statistical analyses. Multivariate analysis was performed using logistic regression (enter method) adjusting by gender, age (adult, child), occupation, type of house, contact with domestic animals, travel history and neighborhoods.

## Results

### Identification of IQT9924 virus as a novel reassortant *Orthobunyavirus*

In 1999, IQT virus strain IQT9924 was isolated in Iquitos, Peru, from a 13-year-old boy who had an illness that included symptoms of fever, headache, eye pain, body pain, arthralgias, diarrhea, and chills. These clinical symptoms were typical of ORO virus infections in Peru [17]. The strain was provisionally identified as ORO virus based on results of serological reactivity in an indirect immunofluorescence test with virus-infected cells.

Sixteen additional IQT9924 virus isolates (provisionally identified as ORO virus) were obtained from febrile patients living in Iquitos: 1 in 2003, 7 in 2005, and 8 in 2006. Of the 17 IQT 9924 virus positive patients, detailed clinical information was available for 16 (Table 2). The most common general symptoms were: chills (16), headache (15), arthralgia or loss of joint function (15), general malaise (14), diminished appetite (13), myalgias (13), retro-orbital pain (11), bone pain (9), and pallor (7). Gastrointestinal symptoms were very common, and 12 patients had at least one of the following symptoms: nausea (10), abdominal pain (7), vomiting (4), or diarrhea (2). No patients had jaundice, hepatomegaly, splenomegaly, abdominal distension, or ascites. Six patients had at least one respiratory symptom: cough (6), rhinorrhea (3), pharyngeal congestion (2), or expectoration (1); no patient had dyspnea. Only one patient complained of a rash, and this was described as erythematous and affecting both the central trunk and distal extremities. With the exception of one patient who had petechiae, none of the patients had hemorrhagic manifestations, including epistaxis, bleeding gums, melena, hematochezia, vaginal bleeding, petechiae, purpura, and ecchymosis. All patients had a fever as this was one of the inclusion criteria.

**Table 2:** Clinical signs and symptoms among patients (n = 16) infected with IQT 9924 virus.

Symptom	Number	Percentage
**General**		
Chills	16	100%
Headache	15	94%
Arthralgia or ↓ joint function	15	94%
General malaise	14	88%
Diminished appetite	13	81%
Myalgias	13	81%
Retro-orbital pain	11	69%
Bone pain	9	56%
Pallor	7	44%
**Gastrointestinal manifestations**	**12**	**75%**
Nausea	10	63%
Abdominal pain	7	44%
Vomiting	4	25%
Diarrhea	2	13%
Jaundice	0	0%
Hepatomegaly	0	0%
Splenomegaly	0	0%
Abdominal distension	0	0%
Ascites	0	0%
**Respiratory manifestations**	**6**	**38%**
Cough	6	38%
Rhinorrhea	3	19%
Pharyngeal congestion	2	13%
Expectoration	1	6%
**Cutaneous manifestations** †	1	6%
**Hemorrhagic manifestations** ‡	1	6%

**†:** The following items were evaluated for this category: Maculopapular rash, erythematous rash, vesicles, and subcutaneous nodules.

**‡:** The following items were evaluated for this category: epistaxis, bleeding gums, melena, hematochezia, vaginal bleeding, petechiae, purpura, and ecchymosis.

Based on virus isolation and serological analyses, the peak incidence for ORO virus was reported in Iquitos as occurring in 2004, 2005 and 2006 [30]. Because the circulation of IQT9924 virus only was detected serologically and genetically (see below) during that time period, we believe that the study most likely reported the incidence of IQT9924 virus.

**Figure 1:**
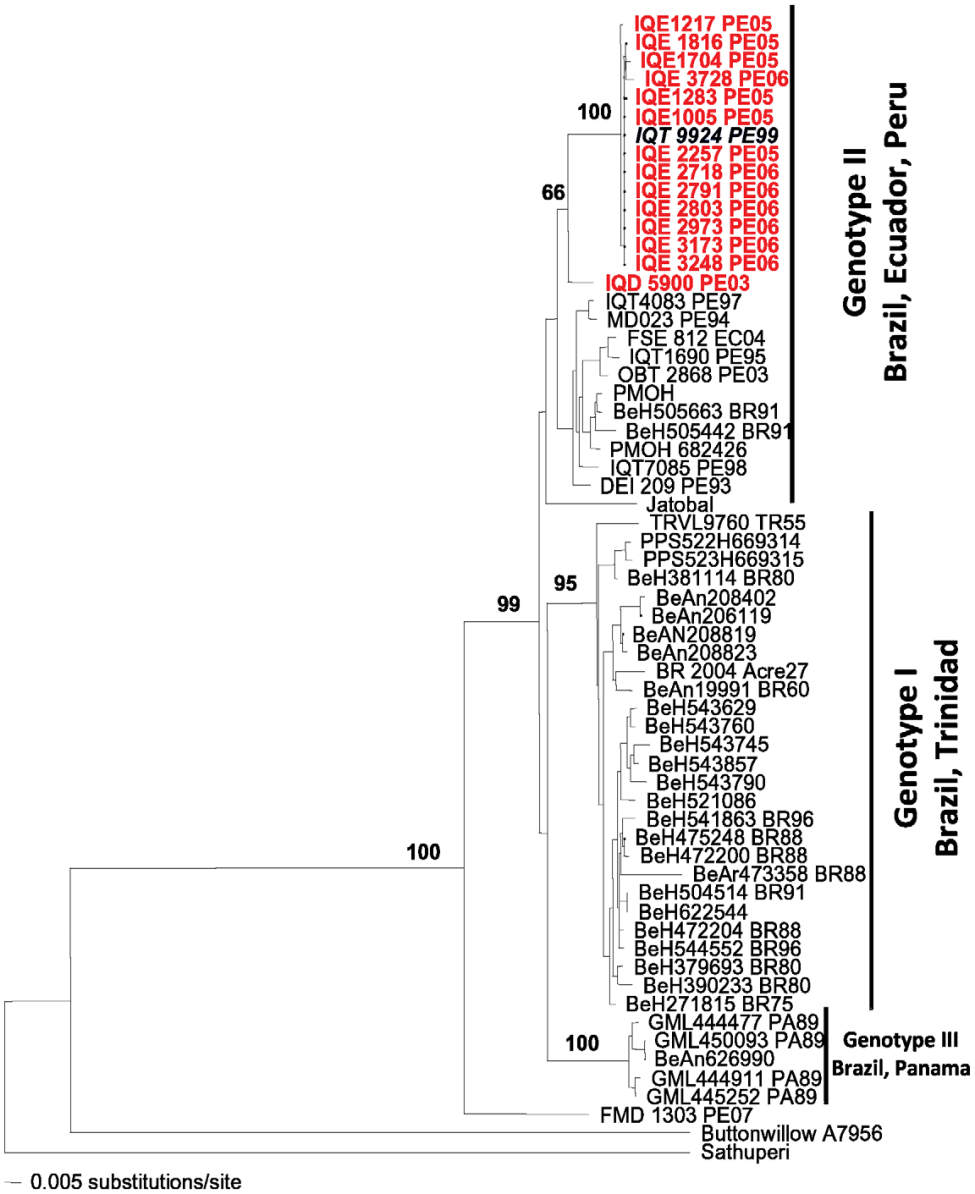
Neighbor joining phylogenetic tree of the Oropouche virus based on the S segment. The tree was derived from recently generated partial nucleoprotein gene sequences of recent ORO-like virus isolates from Peru (depicted in red or italics) and previously published homologous sequences, using the neighbor joining program. Virus strains are labeled by code designation. The tree was rooted using an outgroup comprised of Sathuperi virus. Numbers indicate bootstrap values for the clades to the right.

**Figure 2:**
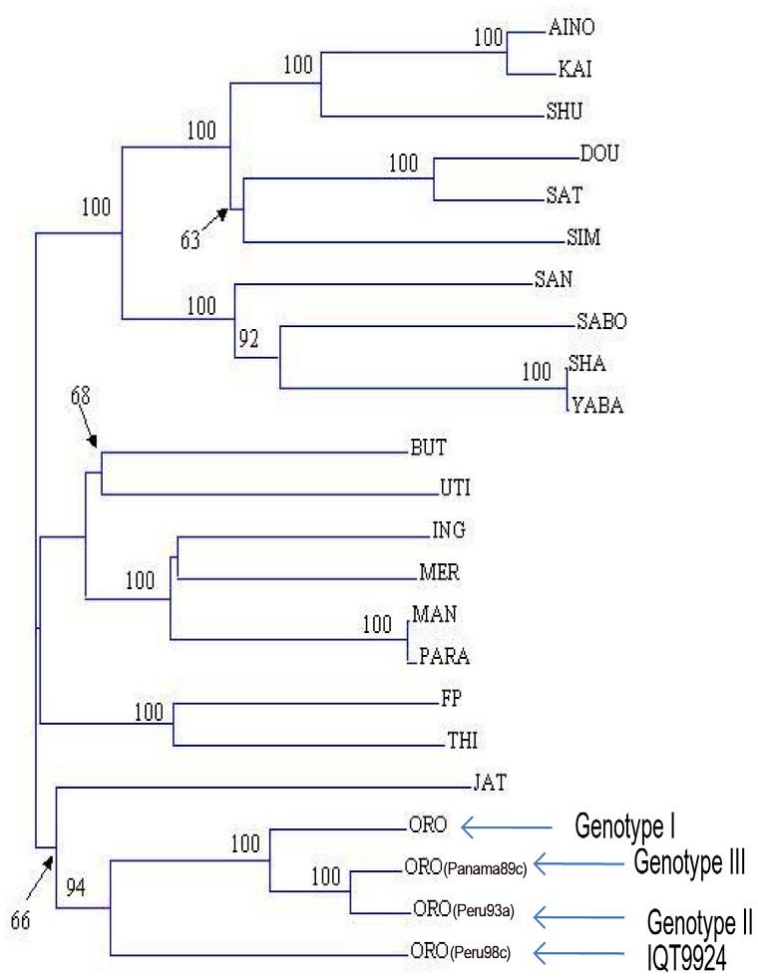
Neighbor-joining phylogenetic tree of the M segment RNA of members of the Simbu serogroup. The tree was constructed based on partial sequences of the Gc gene. Bootstrap values were obtained based on 500 replicates. Virus abbreviations are as follows: Kai, Kaikalur; Shu, Shuni; Dou, Douglas; Sat, Sathuperi; Sim, Simbu; San, Sango; Sha, Shamonda; But, Buttonwillow; Uti, Utinga; Ing, Ingwavuma; Mer, Mermet; Man, Manzanilla; FP, Facey's Paddock; Thi, Thimiri; Jat, Jatobal; ORO, Oropouche.

Genetic analyses of the S, M, and L segments were conducted to determine the relationships of the isolates to ORO virus and other members of the Simbu serogroup. Phylogenetic trees were constructed for both the S- and M-RNA segments based on members of the Simbu serogroup that included an example from each of the three known ORO genotypes [19]. The phylogenetic tree based on the S segment placed the S-RNA of IQT9924 virus among isolates of ORO virus genotype II (Fig. 1), while the M-RNA phylogenetic tree (Fig. 2) indicated that the strain IQT9924 had an M-RNA that was unique from any other Simbu serogroup virus identified to date, but was most closely related to ORO virus. Significantly, IQT9924 virus had an M-RNA distinct from JAT virus, a reassortant with a non-ORO virus M-RNA [6]. The L-RNA phylogenetic tree showed that IQT9924 virus had an L-RNA of ORO virus (data not shown). The M segment fragment sequence (∼590 nucleotides) of IQT9924 displayed only 68% nucleotide and 65% amino acid identity to the prototype ORO strains BeAn 19991 (Brazil) and Tr9760 (Trinidad) whereas the S and L segment fragment sequences exhibited 95% nucleotide and 96% amino acid identity to ORO virus. Based on partial sequences of the S, M and L segments, all ORO-like viruses isolated from patients living in Iquitos after 1999 had similar genetic characteristics and would appear to be examples of IQT9924 virus. Genetic analyses of ORO virus isolates obtained in Peru before 1999, did not reveal the circulation of IQT9924 virus prior to 1999 [19].

### Antigenic characterization of IQT9924

We investigated the serological relationships of IQT9924 virus using cross-neutralization tests. Mouse antisera were prepared against ORO virus strain Trinidad55, IQT9924 virus, and JAT virus. These antisera displayed a 4-fold or greater difference in neutralization titer among these viruses (Table 3), indicating that IQT9924 virus is serologically distinct from ORO and JAT viruses.

**Table 3:** Neutralization of ORO, IQT9924 and JAT virus by mouse antisera.

Mouse Immune Ascitic Fluid			
**Virus**	**Anti-Trinidad 55**	**Anti-IQT9924**	**Anti-JAT**
**Trinidad 55 (ORO)**	>20,480	40	<20
**IQT 9924**	<20	>20,480	<20
**JAT**	<20	<20	1280

Neutralization titers are expressed as the dilution of mouse immune ascitic fluid antiserum inhibiting 80% of plaque forming units.

Next, convalescent sera from patients in Iquitos who had been infected with either OROV or IQT9924 virus were assayed by PRNT to confirm the antigenic differences. A 4-fold or greater difference in neutralization titer was observed between these viruses (Table 4) corroborating our initial findings with mouse antisera. Of particular importance, neutralizing antibody to ORO virus were detected in the acute serum samples of 2 febrile patients from whom the IQT9924 virus had been isolated (Table 5). Furthermore, a boost in ORO neutralizing antibody titers was observed in both patients after infection with IQT9924 (Table 5). Overall, the results suggest that prior ORO virus infection does not protect against febrile disease caused by the new reassortant virus (IQT 9924) and is consistent with the cross-neutralization data indicating that IQT9924 and ORO are distinct viruses, and that there is limited cross-protective immunity such that individuals can be infected and have clinical diseases caused by both viruses.

**Table 4:** Antigenic characterization of ORO and IQT9924 (IQT) viruses.

Convalescent sera	Virus strain	
	IQT 1690 (ORO)	IQT 9924 (IQT)
IQT 4191 (ORO)	40	<40
FSE 813 (ORO)	≥640	<20
IQU 224 (IQT)	<40	≥320
IQE 1922 (IQT)	<20	80
IQE 1936 (IQT)	<20	160
IQE 2391 (IQT)	<20	80
IQE 3811 (IQT)	<20	40
IQE 3409 (IQT)	<20	80
IQE 3473 (IQT)	<20	80

The PRNT assay was done using convalescent sera from febrile patients infected with ORO or IQT9924 (IQT) virus. The PRNT titers are presented as the reciprocal of the highest serum dilution capable of neutralizing 80% of approximately 100 plaque-forming units (PFU) of virus.

**Table 5:** Past infection with ORO virus does not protect against disease with IQT9924 (IQT) virus.

Case	Virus strain	
	IQT 1690 (ORO)	IQT 9924 (IQT)
**Patient 1**IQE 1005 acute	320	<20
IQE 1106 convalescent	≥640	≥640
**Patient 2**IQE 2803 acute	160	<20
IQE3122 convalescent	≥640	320

Two febrile patients had ORO neutralizing antibodies in the acute sera where IQT virus was isolated. The PRNT titers are presented as the reciprocal of the highest serum dilution capable of neutralizing 80% of approximately 100 plaque-forming units (PFU) of virus.

### Hamster virulence of IQT9924

The hamster is used as an animal model of ORO virus infection [36]. Thus, we evaluated the hamster virulence phenotype of IQT9924 virus and compared the results to those obtained with representatives of the three ORO genotypes. The IQT 9924 virus was found to be poorly virulent with a LD_50_ of 4220 PFU and was similar to two Panamanian strains (GML 444477 and GML445252) belonging to ORO genotype III that were non-lethal at the highest doses inoculated (>2,500 and >2,000 PFU, respectively) (Table 6). In comparison, ORO genotype II strain MD023 from Madre de Dios, Peru, was virulent with LD_50_ of 45 PFU. Finally, two genotype I strains, BeH379693 and BeH544552 from Brazil, displayed different virulence phenotypes (>7,000 PFU vs 1 PFU, respectively).

**Table 6:** Hamster virulence of ORO and IQT9924 viruses.

Virus	ORO genotype	Lethality (PFU/LD50)	Average survival time (days)
**BeH379693**	I	>7,000	NA
**BeH544552**	I	1	5.0*
**MD023**	II	45	6.5**
**GML444477**	III	>2,500	NA
**GML445252**	III	>2,000	NA
**IQT9924**	NA	4220	5.5

*at a virus inoculum of 2,000 PFU.

**at a virus inoculum of 14,000 PFU.

NA: Not applicable.

### Viremia levels

Since Simbu serogroup viruses are arboviruses, we undertook preliminary studies with a limited number of serum samples to determine viremia titers among ORO and IQT9924 virus-infected patients. Serum infectivity titers were obtained from a total of 19 patients. Two ORO virus-infected patients had viremias of 6×10^3^ and 7×10^5^ PFU/ml whereas 17 patients infected with IQT9924 virus had levels that were below the limit of detection of the assay (<100) to 1.8×10^5^ PFU/ml (Table 1).

### Risk factors for ORO and IQT9924 virus infection in the city of Iquitos, Peru

Examination of 1037 human serum samples from acute febrile infections in Iquitos revealed that the overall neutralizing antibody prevalence to ORO virus was 14.9% (154/1037) (95% CI 12.8–17.1) whereas prevalence to IQT9924 virus was 15.4% (160/1037) (95% CI 13.3–17.7). Only 3.4% (35/1037) of the serum samples had neutralizing antibodies (>20) to both viruses. Neutralizing antibody prevalence to both ORO and IQT9924 viruses was higher among females than males (17.3% vs 10.2% and 17.5% vs 11.3%, respectively; *p*<0.05).

**Figure 3:**
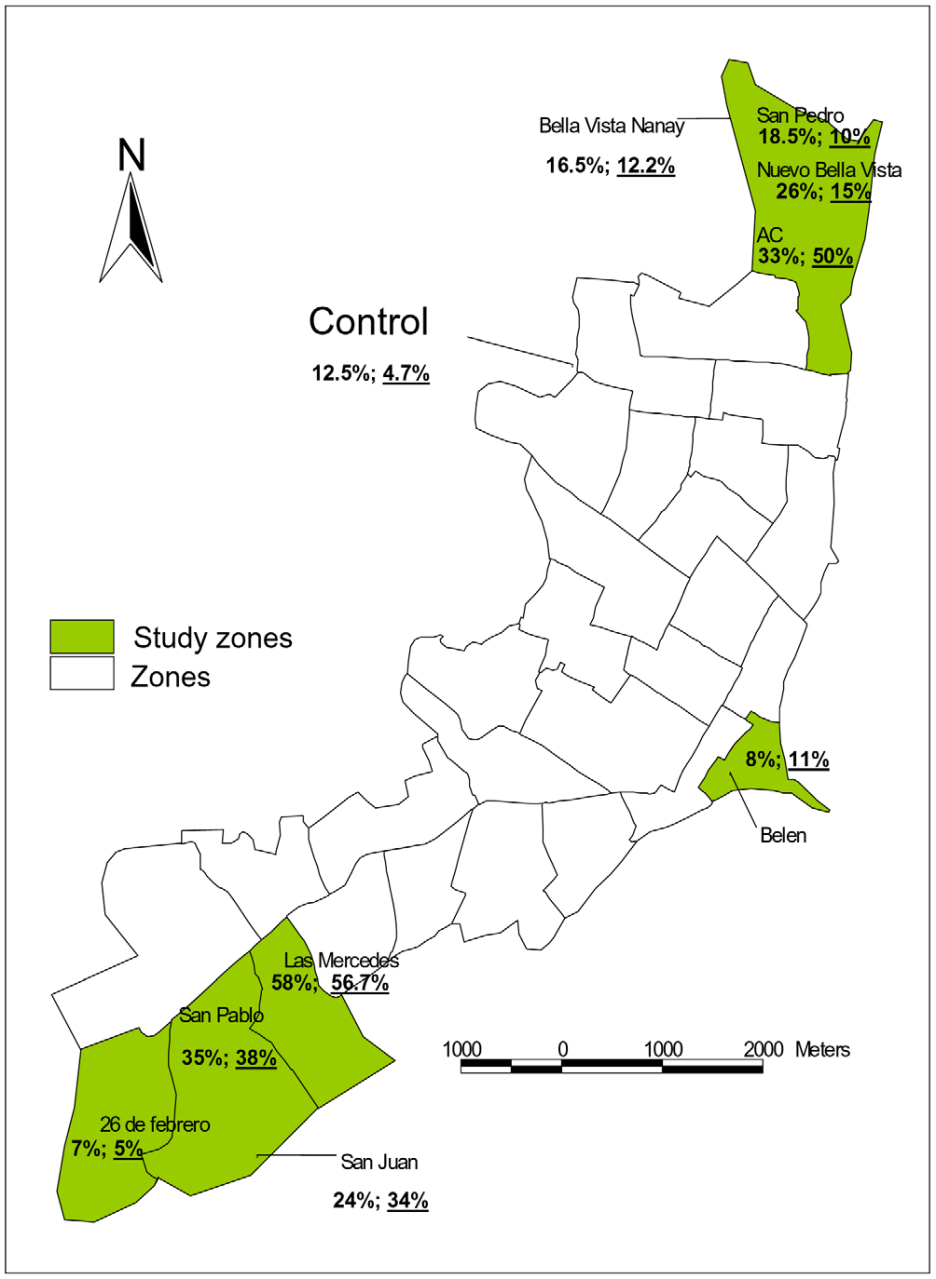
ORO and IQT9924 antibody prevalence among residents from different neighborhoods in Iquitos. ORO virus antibody prevalence is shown in black, underlined numbers represent IQT9924 virus antibody prevalence.

The neutralizing antibody prevalence to ORO virus in persons living in the neighborhoods of San Juan (24.1%) and Bellavista Nanay (16.5%) was higher compared to other neighborhoods (Fig. 3). Similarly, the PRNT antibody prevalence to IQT9924 virus was also higher in the neighborhoods of San Juan (33.9%) and Bellavista Nanay (12.2%). In San Juan, PRNT antibody prevalence to IQT9924 virus was significantly higher than to ORO virus (*p*<0.05). Neutralizing antibody prevalence to both viruses in the study population increased with age after adulthood (0% in 5–9 years old to 32.3% in 40-49 years old for ORO virus and 3.6% in 5–9 years old to 25% in >70 years old for IQT9924 virus) (Table 7). The antibody prevalence to ORO and IQT9924 viruses in adults was 20.1% and 18.8%, respectively, compared to 2.8% and 7.9% in the younger group (<20 years old). The univariate analysis did not detect an association between ORO or IQT9924 virus antibody prevalence and occupation, type of housing, travel or contact with chickens or rodents.

**Table 7:** ORO and IQT virus antibody prevalence rates by age group.

Age group (years)	ORO PRNT_80_		IQT9924 PRNT_80_	
	Positive (%)	Total	Positive (%)	Total
**5–9**	0 (0%)	84	3 (3.6%)	84
**10–14**	4 (3.2%)	126	6 (4.8%)	126
**15–19**	6 (4.4%)	137	19 (13.9%)	137
**20–29**	22 (9.2%)	239	47 (19.7%)	239
**30–39**	42 (22.3%)	188	36 (19.1%)	188
**40–49**	42 (32.3%)	130	20 (15.4%)	130
**50–59**	24 (30.4%)	79	18 (22.8%)	79
**60–69**	11 (28.9%)	38	7 (18.4%)	38
**>70**	3 (18.8%)	16	4 (25%)	16
**Total**	**154 (14.9%)**	**1037**	**160 (15.4%)**	**1037**

Logistic regression models were used to test the significance of each factor. The variables predictive of ORO virus neutralizing antibody prevalence in the model were gender, age and neighborhood, whereas factors such as gender, age, neighborhood, and contact with pigs were predictive of IQT9924 virus antibody prevalence.

## Discussion

The first confirmed ORO fever cases in Peru were reported in 1992 involving a small outbreak of eight febrile cases living in Iquitos [37]. Subsequently, only sporadic cases of ORO fever were reported in Peru, mainly in the Amazon region. The situation in Peru differs from Brazil, where ORO fever outbreaks are usually associated with hundreds of human cases [11], [18], [38]. The reasons for this apparent difference in ORO transmission rates remain unknown. To date, three ORO genotypes have been described based on the nucleoprotein gene (S segment): genotype I among viruses circulating in Brazil and Trinidad, genotype II among viruses from Brazil and Peru, and genotype III among viruses from Brazil and Panama [10], [18], [19]. In this study, we sought to genetically characterize strains from Peru that were provisionally identified as ORO virus. Sequence analyses based on the S and L segments placed the strain IQT9924, isolated in Iquitos in 1999, within ORO genotype II while phylogenetic analyses of the M segment revealed that IQT9924 virus contained a M-RNA segment of a still unidentified Simbu-serogroup virus. Thus, IQT9924 was identified as a Simbu serogroup reassortant virus. Serological characterization of IQT9924 virus confirmed that the virus was distinct from ORO virus. More importantly, we obtained serological evidence that prior ORO virus infection does not protect against clinical disease caused by this new reassortant virus, providing additional confirmation that ORO and IQT9924 are two distinct viral entities. Because most arbovirus laboratories in South America identify ORO virus without detailed serological or genetic characterization, it is uncertain whether IQT9924 circulates in other South American countries. Further genetic and antigenic characterization of ORO isolates from South America is necessary to fully determine the geographic distribution of this newly identified human pathogen.

Studies conducted in Brazil reported viremia titers higher than 3log_10_ suckling mice LD_50_ (SMLD_50_)/ml among ORO- infected patients, including approximately 10% of the patients who developed viremia titers ranging from 5.0 to 5.3 log_10_ SMLD_50_/ml during the first two days of illness [20]. These levels of viremia were high enough to infect *C. paraensis* and transmission was demonstrated to susceptible hosts. Consequently, it has been postulated that humans are the primary amplifying host during ORO fever epidemics [20], [24]. In contrast, viremia levels lower than 5.3log_10_ SMLD_50_/ml were not sufficient to infect *C. paraensis* [24]. In this study, we measured viremia by plaque assay (instead of SMLD_50_) and found that the average viremia levels detected among ORO and IQT9924 virus-infected patients were 4.8±0.7 log_10_ PFU/ml and 3.4±1.1 log_10_ PFU/ml, respectively with at least one ORO-infected patient developing a viremia titer of 5.9 log_10_ PFU/ml (Table 1).

It is not known if *C. paraensis* is the vector of IQT9924 virus. However, studies conducted in and around Iquitos during 1996–1997 identified *C. paraensis* as the most common biting midge of all host-seeking ceratopogonids at 16 sites and *C. insinuatus* being the second most common biting midge [39], [40]. Subsequent studies conducted in 2001, 2002, and 2003 showed that the peaks in biting activities in the Punchana district, located near Iquitos, occurred between October and December for *C. paraensis*. Likewise, peaks in biting activities were observed between October and April for *C. insinuatus* in Santa Clara, near Iquitos [40]. It has not been possible to determine peak incidence rates of ORO fever due to lack of identification of cases. In contrast, data from our febrile surveillance study suggest that the number of IQT9924 virus-infected patients peak from December to April indicating a possible overlap with the biting activities of *C. insinuatus* [30]. Felippe-Bauer *et al* [41] recently identified two new morphological species in the *C. paraensis* complex from the Department of Amazonas and Loreto, Peru. Thus, future studies should investigate the role of this midge in ORO and IQT9924 virus transmission.

In Iquitos, the overall neutralizing antibody prevalence for ORO virus was 14.9% whereas prevalence for IQT9924 virus was 15.4%. These numbers are lower than those from previous studies that reported antibody prevalence to ORO virus (based mostly on IgG antibodies) as high as 35% in certain neighborhoods near Iquitos [14], [15]. Thus, it would appear that ORO virus prevalence varies among neighborhoods and this was demonstrated in our study where neighborhoods, such as San Juan and Bellavista Nanay, have higher ORO virus prevalence rates. Antibody prevalence to ORO and IQT9924 viruses was higher among females and is consistent with previous studies on ORO virus prevalence [15], [42]. Another study carried out in Brazil after an ORO virus outbreak also detected higher antibody prevalence among women [43]; however, a subsequent study in Brazil failed to detect gender differences in attack rates [20].

Our clinical data indicate that the IQT9924 virus shares many of the same clinical manifestations as ORO virus. Headache and chills affected the vast majority of IQT9924-infected patients, similar to previous studies of ORO virus infection [18], [42]. Bone, muscle, and joint pain also were observed commonly for both viruses. Rash and hemorrhagic manifestations have not been classically described with ORO virus infection and were only found in one of 16 IQT9924-infected patients in this study. Interestingly, respiratory complaints—primarily cough—were found in 38% of our IQT9924-infected patients, a finding not commonly reported with ORO virus infection.

Among the *Orthobunyavirus* genus, studies have shown that the pathogenicity of these viruses is multigenic with the M segment being a major determinant [44]. Thus, it is very likely that the donor of IQT9924 virus M segment may also cause human illness in the Amazon region of Peru. It is worth noting that this new virus was first isolated in 1999 and, subsequently in 2005 and 2006, when it was the cause of outbreaks of febrile illness in Iquitos. Some of the patients infected with the IQT9924 virus in 2005 and 2006 resided within Quistococha and Zungarococha, which are considered rural areas near Iquitos. Thus, it is possible that a spill-over of cases occurred from rural to urban areas as was suggested during the 2005-2006 VEEV outbreaks in Iquitos [31]. Unusual high annual river levels occurred in early 2006, which may had an impact on arthropod density and geographic distribution leading to the observed VEEV and IQT9924 virus outbreaks. Finally, it appears that this IQT9924 virus has emerged and possibly replaced ORO virus in Iquitos because ORO virus cases have not been identified in the area since 1999.

In summary, this study identified a new pathogen, IQT9924 a reassortant of ORO virus that was associated with febrile illness in the Amazon region of Peru. We propose the name Iquitos virus for this newly identified *Orthobunyavirus* member of the Simbu-serogroup because there have been cases of disease caused by this virus in the Iquitos area over several years. While reassortment among members of the same serogroup of the *Bunyaviridae* has been identified, few reassortants have been associated with human disease. It will be important to determine the geographic distribution of Iquitos virus and to evaluate its potential as a major public health problem as ORO virus has been done in Brazil.
